# Amygdala enlargement associated with remote epileptogenic lesions

**DOI:** 10.1002/epi.70139

**Published:** 2026-02-05

**Authors:** Horst Urbach, Theo Demerath, Alexander Rau, Dirk‐Matthias Altenmüller, Marcel Heers, Samer Elsheikh

**Affiliations:** ^1^ Department of Neuroradiology, Medical Center – University of Freiburg, Faculty of Medicine University of Freiburg Freiburg Germany; ^2^ Department of Epileptology, Medical Center – University of Freiburg, Faculty of Medicine University of Freiburg Freiburg Germany

**Keywords:** amygdala, epileptogenic lesion, large language model, temporal lobe epilepsy

## Abstract

**Objective:**

To determine the prevalence and possible causes of amygdala enlargement in patients with drug‐resistant temporal lobe epilepsy.

**Methods:**

Patients were retrospectively identified via a radiology information system and a large language model. Magnetic resonance imaging scans were visually re‐analyzed and amygdala volumetry applied.

**Results:**

The term “amygdala” was used in 89 of 1853 patients. Of those, 54 had lesions in the amygdalae, 20 had isolated amygdala enlargements, and 15 patients had amygdala enlargements and remote epileptogenic lesions. Objective processing of imaging data confirmed higher amygdala volumes of both latter groups (2.09 ± 0.28 mL, 2.23 ± 0.33 mL vs 1.56 ± 0.22 mL).

**Significance:**

When amygdala enlargement occurs with remote epileptogenic lesions and patients become seizure‐free after remote lesion resection, amygdala enlargement is likely the consequence of seizures, but not their cause. In addition, isolated amygdala enlargements can be the consequence of epileptic seizures.

**Key points**

- Amygdala enlargement with homogenous signal can be the cause or the consequence of seizures.
- When patients become seizure‐free following resection of a remote epileptogenic lesion, amygdala enlargement is likely the consequence of seizures.
- Large language models facilitate the retrospective analysis of large data sets.

## 1 INTRODUCTION

Amygdala enlargement on magnetic resonance imaging (MRI) has been reported in 4% of patients with drug‐resistant temporal lobe epilepsy (TLE).^1^ It has been considered a distinct electroclinical syndrome, a pathological epileptogenic substrate for otherwise non‐lesional TLE, a substrate of an (autoantibody‐negative) limbic encephalitis, or it may occur contralateral to the epileptogenic zone in patients with hippocampal sclerosis.^2^, ^3^, ^4^, ^5^, ^6^, ^7^, ^8^, ^9^, ^10^ Normal histopathological findings in >80% of patients could also suggest that amygdala enlargement is a secondary reactive process to seizures in the epileptogenic temporal lobe.^11^ The purpose of this study was to determine the prevalence and possible causes of amygdala enlargement in patients with drug‐resistant temporal lobe epilepsy. We hypothesized that a percentage of patients with epilepsy would carry epileptogenic lesions remote from the enlarged amygdala and that amygdala enlargement regresses, when patients become seizure‐free after epilepsy surgery.

## 2 METHODS

### 2.1 Patient selection

Patients were retrospectively identified via a radiology information system (RIS) (RadCentre, Mesalvo, Mannheim, Germany) and a large language model. We employed the Qwen3‐30B‐A3B model (https://arxiv.org/abs/2505.09388), accessed via the Ollama framework (https://ollama.com), to process radiological reports written in German. We used the Mixture‐of‐Experts architecture with ~30 billion parameters in total, which is optimized for multilingual natural language understanding, including German. All inference was conducted locally, in zero‐shot inference mode without additional fine‐tuning.

Search criteria were referral from a tertiary epilepsy center, a head MRI scan with an epilepsy‐dedicated protocol on a 3 Tesla scanner (Magnetom Prisma, Siemens Healthineers, Erlangen, Germany),^12^ and a report including information on the “amygdala.”

Further workup included electroencephalography (EEG), noninvasive video‐EEG monitoring (VEM), and invasive VEM with either a combination of subdural electrodes and few intracerebral depth electrodes or stereoelectroencephalography (SEEG) with multiple intracerebral depth electrodes in a subgroup of patients (see Tables 1 and 2). When applied, depth electrodes covered the amygdala and hippocampus in all patients.

**TABLE 1:** Isolated amygdala enlargement (AE).

#	Age, sex	Seizure types	Seizure onset	Lateralization and localization by semiology	Work‐up	Interictal epileptiform potentials	Ictal seizure patterns	Therapy and outcome
1	49, m	FPC, FIC	déjà vu, fear, olfactory and gustatory sensation	left T	VEM, PET	left T	N/A	drug therapy
2	44, m	FPC, FIC	fear, epigastric and gustatory sensation, paraesthesias	left T	VEM, biopsy: gliosis	left T	left hemisphere	drug therapy, seizure‐free (Engel IA, Wieser 1), regression of AE
3	26, f	FPC, FIC, FBTC	cephalic sensation	left T	VEM	left T	left T	drug therapy
4	33, f	FPC, FIC	epigastric and cephalic sensation	right T	SEEG	right temporal pole, amygdala, hippocampus	right temporal pole, amygdala, hippocampus	drug therapy
5	31, f	FIC	N/A	N/A	EEG	N/A	N/A	drug therapy, regression of AE
6	27, m	FPC, FIC, FBTC	déjà vu, cephalic sensation	right T	VEM	right T > left T	right T	drug therapy seizure‐free (Engel IA, Wieser 1)
7	28, f	FPC, FIC, FBTC	epigastric sensation	none	VEM	right T	right T > left T	drug therapy, seizure‐free (Engel IA, Wieser 1), no regression of AE
8	15, f	FIC, FBTC	no aura	left T	VEM	left T	left T	drug therapy, SEEG scheduled
9	21, m	FPC, FIC, FBTC	thoracic sensation	left T	SEEG	left entorhinal cortex, amygdala, hippocampus	left entorhinal cortex, amygdala, hippocampus	lesionectomy, histology: gliosis, seizure‐free (Engel IA, Wieser 1)
10	56, m	FPC, FIC	visual	none	VEM	left T > right T	left T, left TO	drug therapy
11	51, f	FPC, FIC, FBTC	déjà vu	right T	SEEG	right temporal pole, amygdala, hippocampus	right hippocampus	ATR inclusive AHE, histology: gliosis, seizure‐free (Engel IA, Wieser 1)
12	32, f	FPC, FIC, FBTC	epigastric and cephalic sensation	left T	SEEG	left temporal pole, entorhinal cortex, amygdala, hippocampus	left temporal pole, entorhinal cortex	ATR without AHE, histology: gliosis, Engel IVB, Wieser 5
13	23, m	FPC, FIC, FBTC	auditory sensation	left T	subdural and depth electrodes	multifocal left T	left superior temporal gyrus	ATR inclusive amygdala, histology: gliosis, Engel IVB, Wieser 5
14	60, f	FPC, FIC, FBTC	epigastric sensation	N/A	VEM	right T	N/A	drug therapy, seizure‐free (Engel IA, Wieser 1)
15	24, m	FPC, FIC, FBTC	déjà vu, epigastric sensation	none	VEM	right T	right T	drug therapy
16	40, m	FBTC	no aura	N/A	VEM	right T	N/A	drug therapy
17	27, m	FPC, FIC, FBTC	epigastric sensation, paraesthesias	left T	VEM	left T	left T	drug therapy
18	38, m	FPC	déjà vu, epigastric and olfactory sensation	none	VEM	left T	left T	drug therapy
19	78, f	FPC, FIC, FBTC	epigastric sensation	N/A	VEM	left T	N/A	drug therapy
20	40, f	FPC, FIC, FBTC	epigastric and cephalic sensation	right T	SEEG	right temporal pole, hippocampus; left T	right temporal pole, entorhinal cortex, amygdala	ATR inclusive amygdala, histology: gliosis, Engel IB, Wieser 2

Abbreviations: AE, amygdala enlargement; AHE, amygdalohippocampectomy; ATR, anterior temporal lobe resection; FBTC, focal to bilateral tonic clonic seizures; FIC, focal impaired consciousness seizures; FPC, focal preserved consciousness seizures; N/A, not available; SEEG, stereo EEG; T, temporal; TO, temporo‐occipital; VEM, non‐invasive video‐EEG monitoring.

**TABLE 2:** Amygdala enlargement (AE) and remote epileptogenic lesions.

#	Age, sex	Remote lesion	Seizure types	Seizure onset	Lateralization and localization by semiology	Work‐up	Interictal epileptiform potentials	Ictal seizure patterns	Therapy and outcome
21	23, f	atrophy of fronto‐orbital gyri	FPC, FIC, FBTC	epigastric sensation	right T	SEEG	right multifocal frontal, temporo‐polar, amygdala	right inferior frontal gyrus	2 frontal lobe resections, histology: gliosis, FCD IIA, Engel IVA, Wieser 4
22	56, f	postsurgical defect after SAH and MCA aneurysm clipping	FPC, FIC, FBTC	thoracic sensation	right T	VEM	right T	right T	drug therapy
23	59, m	nodular periventricular heterotopias right > left	FBTC	N/A	N/A	VEM	right T	N/A	drug therapy
24	48, w	cavernoma right parahippocampal gyrus	FPC, FBTC	vegetative symptoms	right T	VEM	right T	right T	resection
25	23, f	cavernoma left parahippocampal gyrus	FPC, FIC, FBTC	epigastric sensation	left T	VEM	left T	left T	drug therapy
26	46, m	frontobasal contusions	FPC, FIC	indescribable sensation	left T	SEEG	left temporal pole, entorhinal cortex amygdala, hippocampus, posterior middle temporal gyrus	left amygdala, hippocampus	ATR inclusive AHE, histology: gliosis, seizure free (Engel IA, Wieser 1)
27	36, m	resected FCD left frontal lobe	FPC, FIC, FBTC	paraesthesias	left F	VEM	left FT	Left FCP	new resection frontal lobe scheduled
28	34, f	bilateral temporo‐basal encephaloceles obesity, 18 cm H20	FIC, FBTC	no aura	T non lateralized	VEM	right T, left T	right T, left T, not lateralized	drug therapy
29	35, m	right‐side hemiatrophy and parietal lesion	FPC, FIC, FBTC	déjà vu, paraesthesias	right T	VEM	right FT, left T	right T, FT	CSF‐ab and PET scheduled
30	43, m	cavernoma right fusiform gyrus	FIC, FBTC	no aura	right T	VEM	right T	right T	resection, seizure free (Engel IA, Wieser 1)
31	23, m	FCD II right supramarginal gyrus	FPC, FIC, FBTC	vertigo, out‐of‐body experience	right P	VEM	right FCTP	right FCTP	drug therapy
32	40, m	cavernoma right superior temporal gyrus	FBTC	no aura	N/A	EEG	N/A	N/A	resection, seizure free (Engel IA, Wieser 1), regression of AE
33	33, f	nodular periventricular heterotopias	FPC, FIC	déjà vu	N/A	EEG	N/A	N/A	drug therapy
34	22, f	ganglioglioma right inferior temporal gyrus	FPC, FIC	déjà vu	right T	VEM	right T	right T	resection, seizure free (Engel IA, Wieser 1), regression of AE
35	42, f	enzephalocele right temporal basal, IIH, 55 cm H20	FPC, FBTC	epigastric sensation, fear	N/A	VEM	right T	N/A	drug therapy

Abbreviations: AE, amygdala enlargement; AHE, amygdalohippocampectomy; ATR, anterior temporal lobe resection; CSF‐ab, cerebrospinal fluid antibodies; F, frontal; FBTC, focal to bilateral tonic clonic seizures; FCP, fronto‐centro‐parietal; FCTP, fronto‐centro‐temporo‐parietal; FIC, focal impaired consciousness seizures; FPC, focal preserved consciousness seizures; FT, fronto‐temporal; N/A, not available; SEEG, stereo EEG; T, temporal; VEM, non‐invasive video EEG Monitoring.

### 2.2 Visual MRI analysis

MRI scans were reviewed secondarily by a neuroradiologist (H.U.) to ascertain that the amygdala was enlarged and the T2/fluid‐attenuated inversion recovery (FLAIR) signal was homogenous and normal or only slightly increased.

### 2.3 Volumetric analysis

In order to prove amygdala enlargement, whole amygdala volumetry was applied.

Three‐dimensional (3D) T1‐weighted 1.0 mm isotropic MRI data were processed with FreeSurfer V6.0.^13^, ^14^ The total intracranial volume (TIV) and the amydgala volume after parcellation according to the Desikan–Killiany atlas were read out, and each segmentation was visually inspected for quality control.

Amygdala volumes of the affected and contralateral sides and TIV were compared to a group of 66 age‐matched healthy controls.

Seizure semiology and clinical information were recorded from the patient's charts and reviewed by an epileptologist (D.M.A.). Autoimmune inflammation was ruled out clinically using the Graus criteria.^15^

### 2.4 Statistical analysis

Amygdala volumes of the visually identified ”affected” and of the contralateral sides were compared using analyses of covariance (ANCOVAs) controlling for age, sex, and the TIV with Tukey's post hoc test. For this, the mean of both amygdalae was employed. In addition, we compared the volumes of affected vs contralateral sides within the amygdala enlargement group using paired *t* tests. A *p*‐value < 0.05 was considered statistically significant.

### 2.5 Ethics

The study was approved by the local ethics committee (217/17). Due to the retrospective design, informed consent was waived.

## 3 RESULTS

**FIGURE 1:**
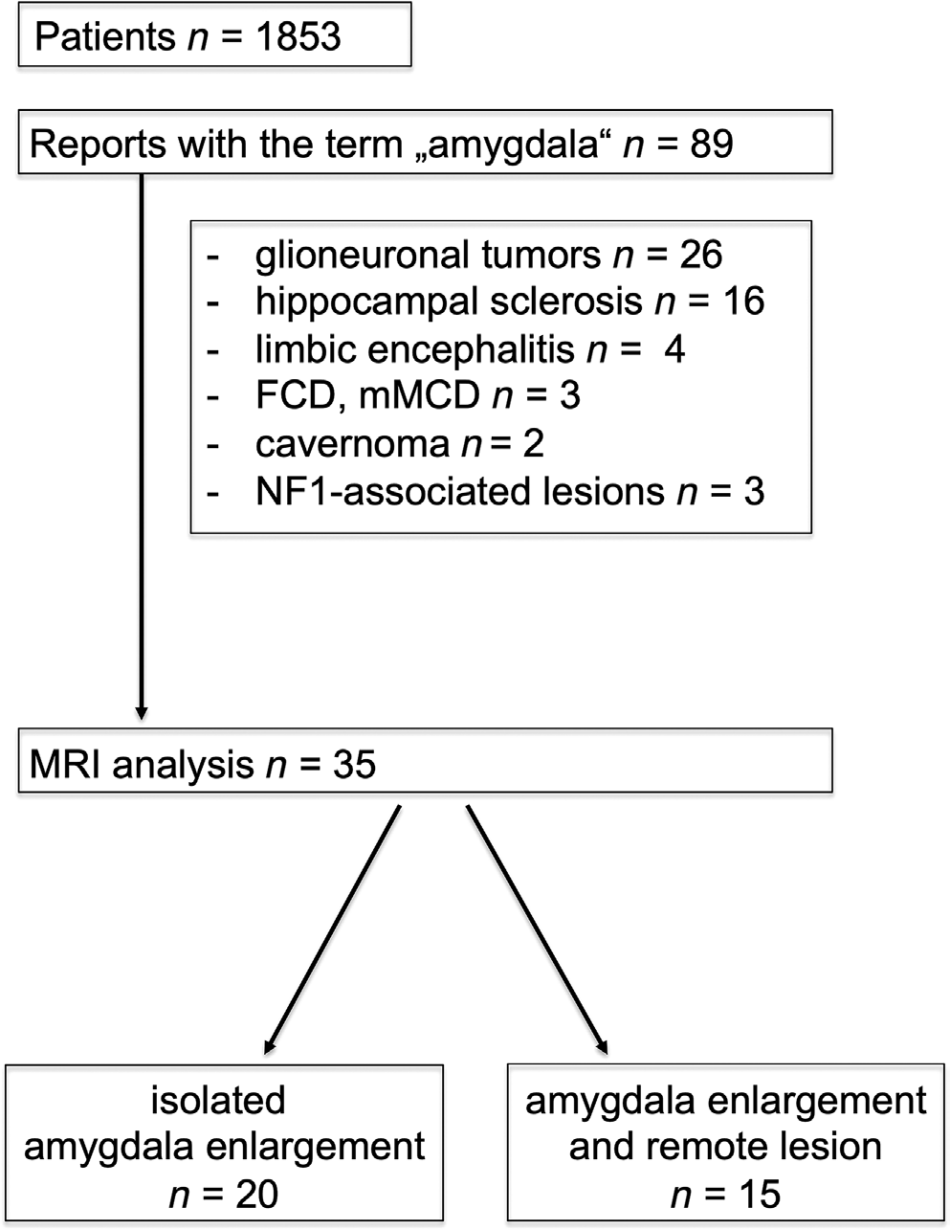
Study flow chart.

Our search delivered 2875 reports belonging to 1853 patients between 2016 and 2024. Eighty‐nine reports in 1853 patients used the term “amygdala” (4.8%). The majority had glioneuronal tumors (29%) or hippocampal sclerosis (18%), whereas limbic encephalitis (4%) and focal cortical dysplasia / mild malformation of cortical development (FCD/mMCD) (3%) were rather rare diagnoses (Figure 1).

**FIGURE 2:**
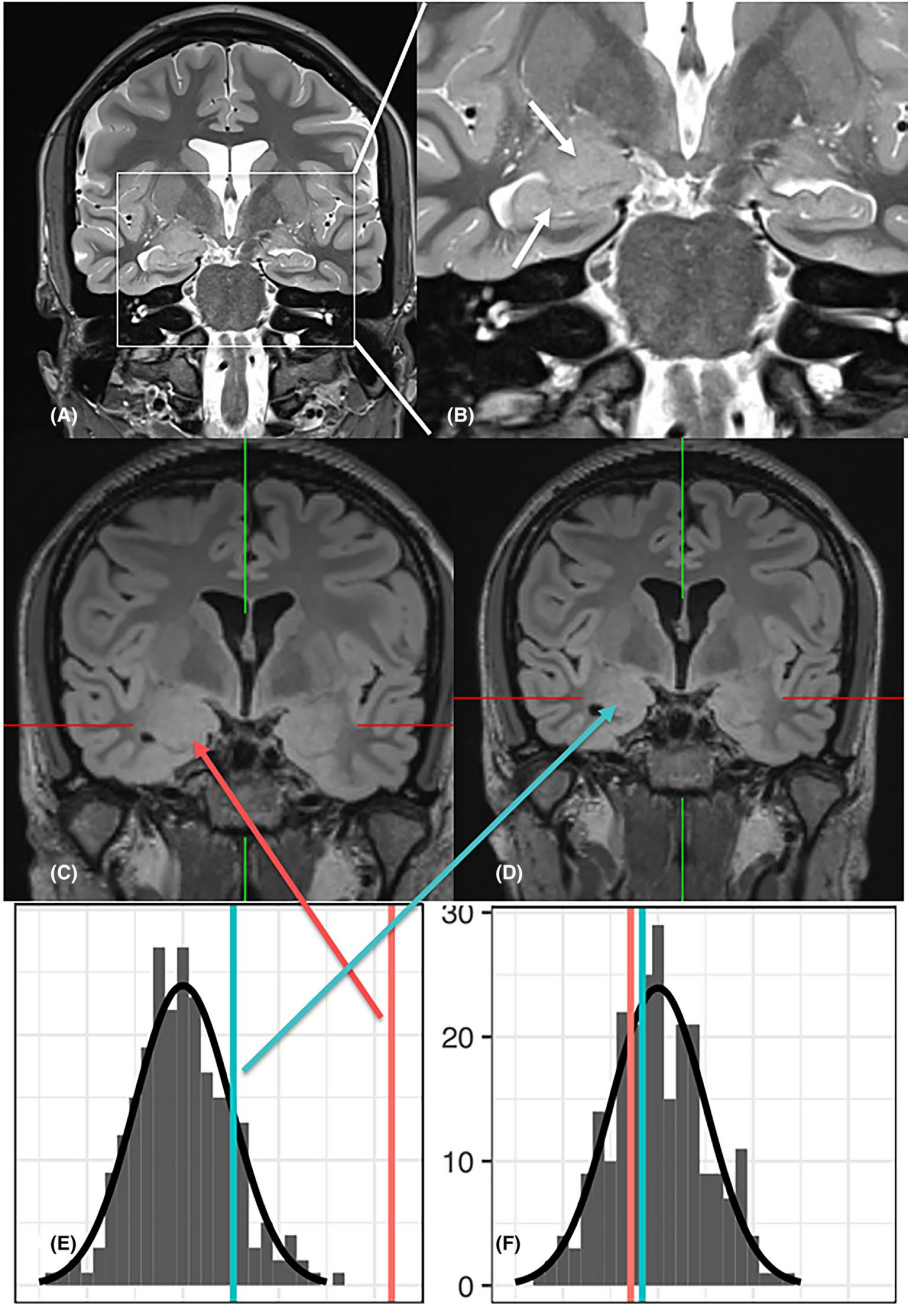
Isolated amygdala enlargement in a 31 year‐old woman (#5) with focal seizures with preserved and impaired consciousness. The right amygdala and the hippocampal head are clearly enlarged (FreeSurfer volumetry: *Z*‐score > 4). A follow‐up scan 6 months later shows regredient amygdala volume (*z*‐score 1).

**FIGURE 3:**
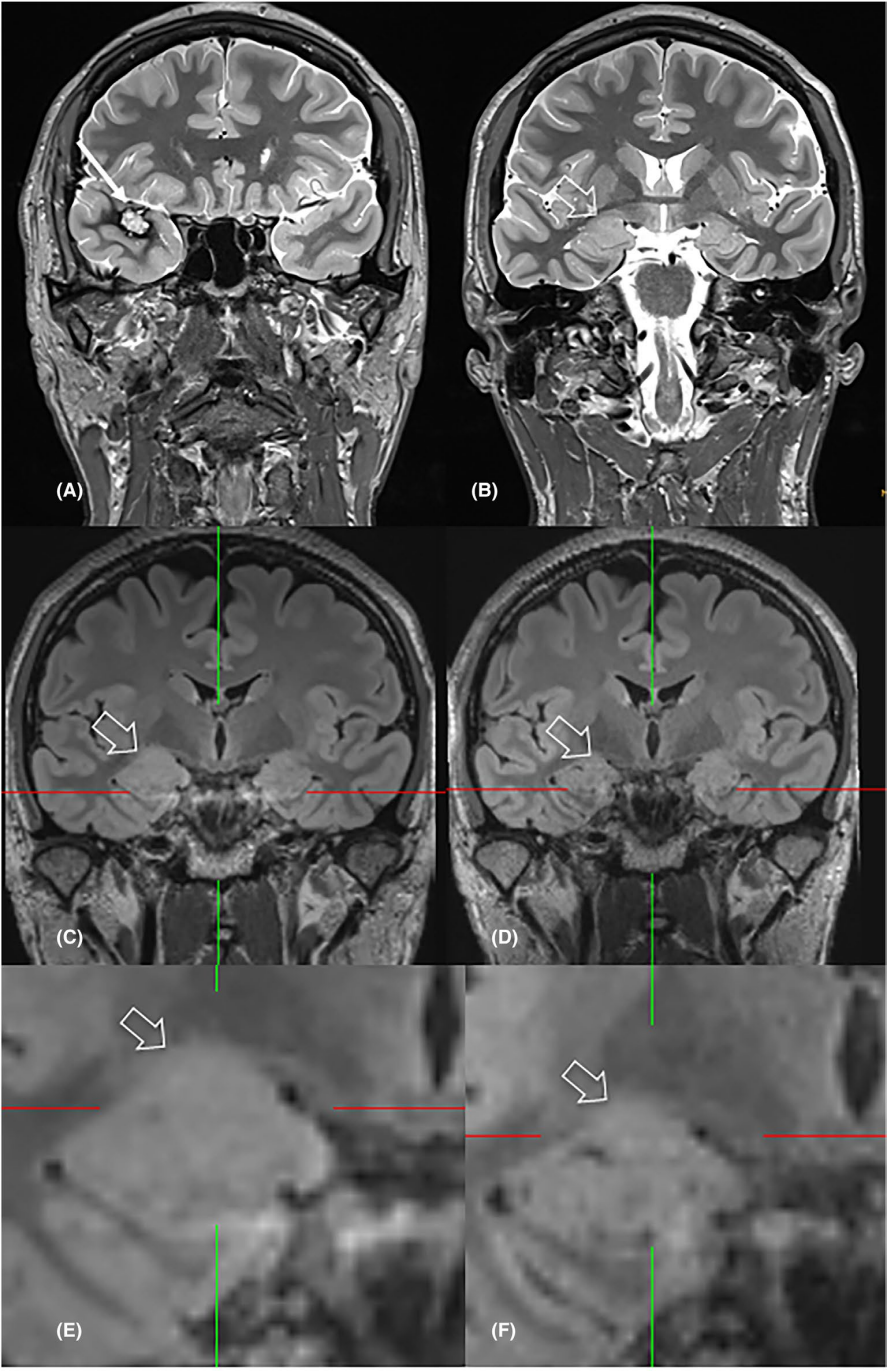
A 40 year‐old man (#32) presented with bilateral tonic clonic seizures. MRI revealed a cavernoma of the right superior temporal gyrus (A, C: Arrow) and right‐sided amygdala enlargement (B, D: Hollow arrow). Three months after cavernoma resection (E: Arrow) the patient was seizure‐free and amygdala enlargement had regressed (F: Hollow arrow).

**FIGURE 4:**
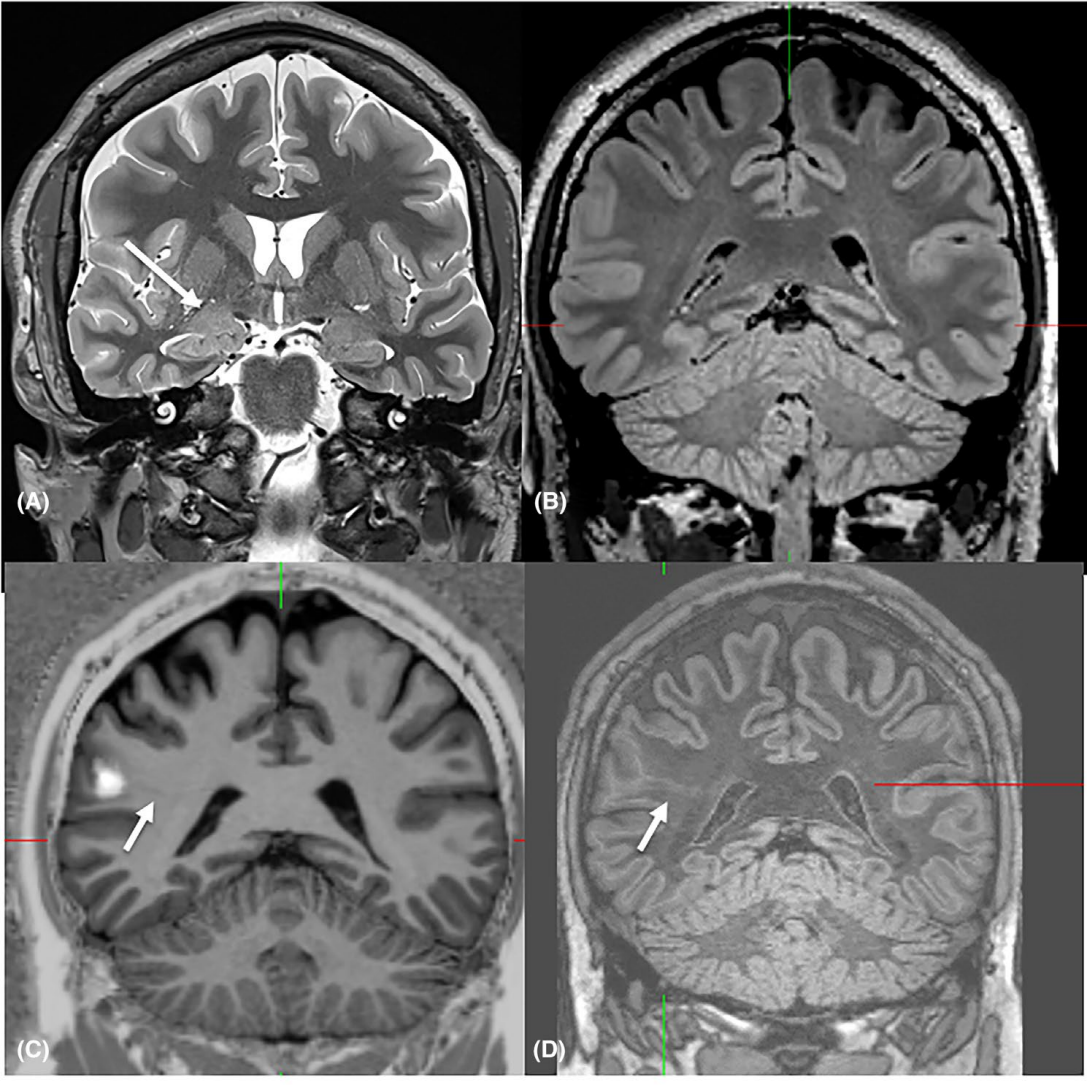
A 23‐year‐old man (#31) presented with right‐sided drug‐resistant focal seizures since the age of 7. Several reports had described right‐sided amygdala enlargement (A: Arrow). A FCD 2 with a transmantle sign (C, D: Arrow) was only identified after voxel‐based postprocessing of the MP2RAGE data set (C) and confirmed on the FLAWS sequence (D).

**FIGURE 5:**
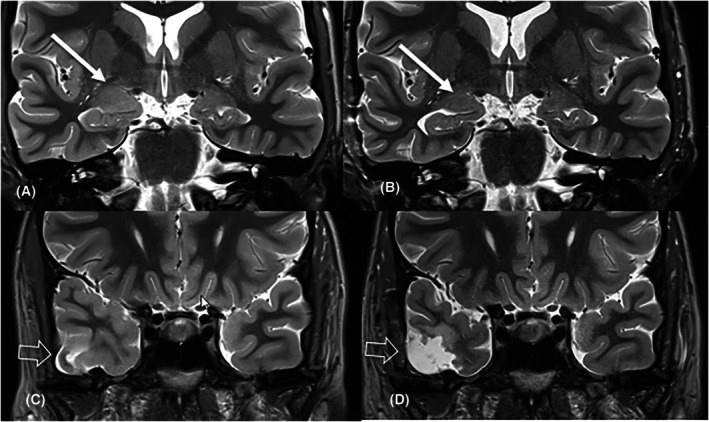
A 22 year‐old woman (#34) presented with focal seizures with preserved and impaired consciousness. MRI showed right‐sided amygdala enlargement and a ganglioglioma of the inferior temporal gyrus. Six months after resection she was seizure‐free and amygdala enlargement had regressed.

Twenty patients (22%) had isolated amygdala enlargement (Figure 2), and another 15 patients (17%) had amygdala enlargement and remote epileptogenic lesions (Tables 1 and 2), (Figures 3, 4, 5).

All amygdala enlargements in both latter groups were on the side from which seizures originated and the amygdala signal was homogenous and slightly hyperintense. The enlargement involved the hippocampal head in 12 patients (Figure 2).

**FIGURE 6:**
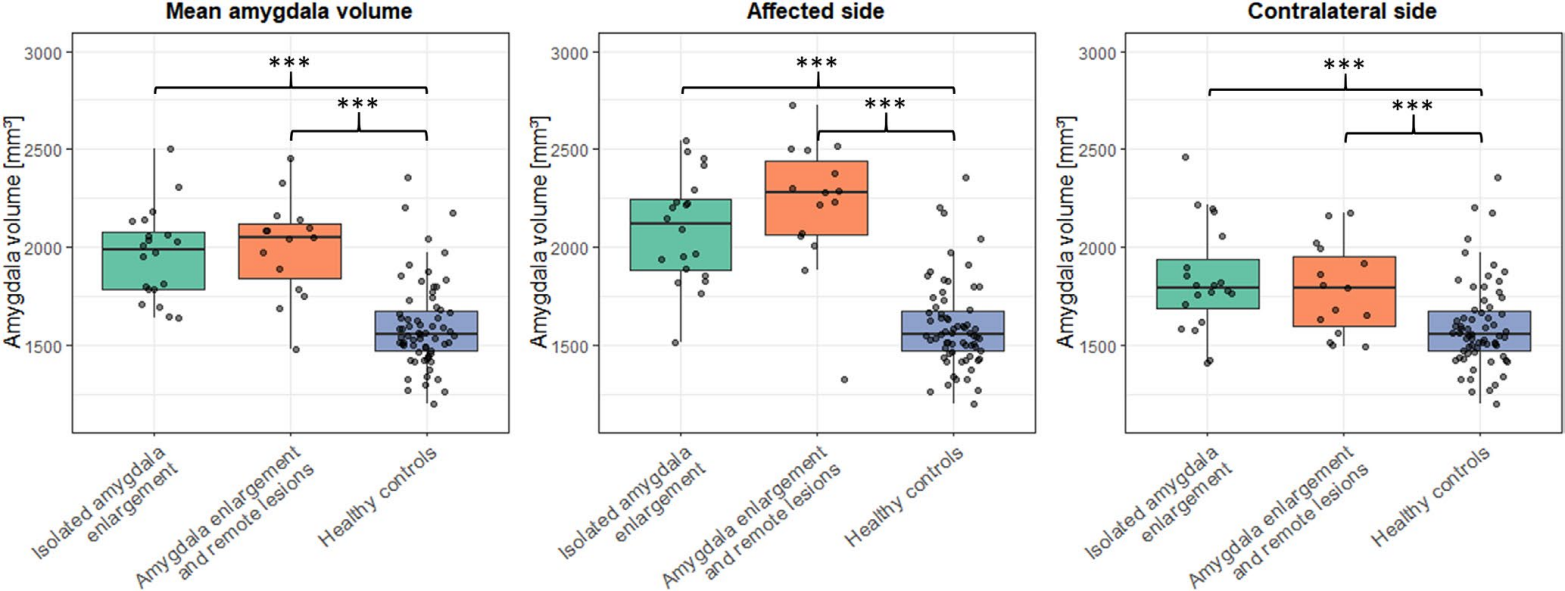
Amygdala volumes of patients with isolated enlargement as well as of patients with remote lesion are larger than in healthy controls. ***p < 0.001.

Amygdala volumes of the affected sides were significantly larger than the mean of both amygdalae in healthy controls (healthy controls 1.56 ± 0.22 mL, isolated amygdala enlargement 2.09 ± 0.28 mL, *p* < 0.001; amygdala enlargement with remote lesion 2.23 ± 0.33 mL, *p* < 0.001; Figure 6). I addition, the unaffected contralateral amygdalae were larger than in healthy controls (1.83 ± 0.23 mL, *p* < 0.001; 1.78 ± 0.27 mL, *p* = 0.008); still the affected sides were significantly larger than the contralateral sides (*p* < 0.001).

Invasive VEM with depth electrodes was performed in 6 of 20 patients with isolated amygdala enlargements; it showed ictal amygdala discharges in three patients (Patients 4, 9, and 20). Five of these patients underwent surgical therapy (anterior temporal lobe resection with [Patients 11, 13, and 20) or without [Patient 12] resection of the amygdala and resection of amygdala and anterior hippocampus in one patient [Patient 9]), and had an unrevealing histology. Two patients became seizure‐free following surgery (Patients 9 and 11) and three did not (Patients 12, 13, and 20), (Table 1).

Fifteen patients received drug therapy without epilepsy surgery. Amygdala enlargement was shown to regress in two patients (Patients 2 and 5), and remained unchanged in another patient (Patient 7) (Figure 2; Table 1).

With regard to the group with remote potentially epileptogenic lesions, SEEG was performed in two patients (Patient 21 and 26). The amygdala was resected in one patient after ictal seizure patterns initially involving the amygdala but not the lesional fronto‐orbital cortex were shown (Patient 26). In total, the remote lesions were resected in five patients (Patients 21, 24, 30, 32, and 34), three of them became seizure‐free (Patients 30, 32, and 34); in two of them regression of amygdala enlargement was proven on follow‐up MRI (Patients 32 and 34). Of note, five remote lesions had been overlooked on previous MRI scans (Patients 21, 26, 28, 31, and 34; Figure 4).

## 4 DISCUSSION

This large language model driven study searching for the term amygdala in patients referred from a tertiary epilepsy center for epilepsy‐dedicated MRI shows that a significant portion of patients with amygdala enlargement (~17%) had “remote” potentially epileptogenic lesions, often within the ipsilateral temporal lobe. When these patients become seizure‐free following resection of the remote epileptogenic lesion, but not the amygdala, or when the amygdala enlargement regresses afterwards, amygdala enlargement is likely the consequence of seizures (Patients 32 and 34; Figures 3 and 5). In contrast, when patients show ictal amygdala discharges on invasive VEM with depth electrodes and become seizure‐free following resection of the amygdala, the enlarged amygdala is considered the (or part of the) epileptogenic lesion.

In 20 patients (~22%), no epileptogenic lesion was found. Thus these patients are considered to have isolated amygdala enlargement with the visual impression confirmed by FreeSurfer‐based volumetry. Of interest, compared to healthy controls and adjusted for age and sex, amygdala volumes in both groups were also larger on the contralateral side; still the “affected” side was significantly larger than the contralateral side. In these patients, both conditions are possible: The amygdala enlargement is the epileptogenic lesion as proven by SEEG and seizure freedom following surgery in two of five patients (Patients 9 and 20). Or amygdala enlargement is the consequence of seizures as indicated by amygdala enlargement regression in two of three patients with drug therapy and available follow‐up volumetric data (Patients 2 and 5).

Patients with amygdala enlargement regression are known to have better seizure free outcomes compared to those with stable MRI findings. In a prospective observational study, five of six (83%) compared to six of 25 (24%) patients with persistent amygdala enlargement got seizure‐free.^10^ Regression of amygdala enlargement could be related to a reduction of the frequency of epileptogenic seizures or (a different) antiseizure medication (Figure 2), however, the definite cause remains unclear. In five patients with isolated amygdala enlargement, surgery was performed but did not reveal a histological abnormality beyond the finding of gliosis. Amygdala enlargement associated with hippocampal gliosis also belongs to this group, although amygdala enlargement is less prominent and was not identified via the large language model in this study.^16^, ^17^ While the amygdala can be enlarged in patients with hippocampal gliosis, the ipsilateral amygdala is typically smaller, when patients have hippocampal sclerosis.^16^ However, hippocampal gliosis, also denominated as “no hippocampal sclerosis/ gliosis only” is a rather uncertain histopathological diagnosis as there is no neuronal loss but only the qualitative metrics “increased GFAP staining”.^18^

## 5 LIMITATIONS

We easily could have missed subtle amygdala enlargement since patients were selected via a large language model requiring that the term amygdala was used in the report and an enlarged amygdala visually identified. Amygdala volumetry is superior to visual analysis, subtle enlargements may be missed in ≈ 40% of patients.^19^

When a typically epileptogenic lesion was identified, concomitant amygdala enlargement could have been overlooked or not reported, a diagnostic error type classified as “search of satisfaction”.^20^

The amygdala is often enlarged when patients have (autoimmune) limbic encephalitis, in many patients enlargement is bilateral.^7^, ^8^, ^9^ However, as CSF was not investigated in all patients, patients with autoimmune encephalitis could have been falsely classified.

In further patients with isolated amygdala enlargements, the cause of amygdala enlargement remains speculative. Both, a distinct electroclinical syndrome of temporal lobe epilepsy or a non‐specific finding outside the primary seizure focus have been discussed.^6^, ^21^, ^22^

Finally, due to the search via a large language model we were not able to control for possible clinical confounders such as drug‐resistance, seizure frequency, and/or medication history.

## 6 CONCLUSION

Amygdala enlargement with homogenous signal can be the cause or the consequence of seizures. Isolated amygdala enlargement is more common but amygdala enlargement with remote epileptogenic lesions does occur. When patients get seizure‐free following resection of a remote epileptogenic lesion, but not the amygdala, or when amygdala enlargement regresses after remote surgery or with drug therapy, amygdala enlargement is likely the consequence of seizures. The fact that some patients had “remote” and “overlooked” epileptogenic lesions underlines the uncertainty of the diagnosis “isolated amygdala enlargement” and the need of epilepsy‐dedicated MRI including postprocessing.

## AUTHOR CONTRIBUTIONS

HU concept, data analysis, editing, final proof TD data analysis, final proof, AR data analysis, statistics, final proof, DMA, MH data analysis, editing, final poof, SE data analysis, final proof.

## FUNDING INFORMATION

No funding.

## CONFLICT OF INTEREST STATEMENT

HU received honoraria for lectures from Bayer, Biogen, GE, Eisai, Mbits, Lilly, and is coeditor of Clin Neuroradiol, TD is consultant for Medtronic and received travel and educational grants from Stryker, Balt, Medtronic. MH received travel grants from Jazz/GW Pharmaceuticals and Precisis, and honoraria for lectures from Eisai and Arkana. SE received Research grants from, Bracco, Medtronic and honoraria for lectures from Penumbra, Medtronic, and Microvention. AR and DMA have nothing to disclose.

## ETHICAL PUBLICATION STATEMENT

We confirm that we have read the Journal's position on issues involved in ethical publication and affirm that this report is consistent with those guidelines.

## Data Availability

Data are available upon personal request by the corresponding author.
